# Association of Obstructive Sleep Apnea with Asthma: A Meta-Analysis

**DOI:** 10.1038/s41598-017-04446-6

**Published:** 2017-06-22

**Authors:** De-Lei Kong, Zheng Qin, Hui Shen, Hong-Yu Jin, Wei Wang, Zan-Feng Wang

**Affiliations:** Department of Respiratory Disease, The First Affiliated Hospital of China Medical University, Shenyang, Liaoning 110001 China

## Abstract

This study evaluates the relationship between obstructive sleep apnea (OSA) and asthma. Literature search was carried out in several electronic databases and random effects meta-analyses were performed to obtain pooled estimates of the prevalence of OSA, OSA risk and sleep disordered breathing (SDB) in asthma patients and pooled odds ratios of the prevalence between asthma and non-asthma patients. In adult asthma patients, the prevalence [95% confidence interval] of OSA, OSA risk, and SDB was 49.50 [36.39, 62.60] %, 27.50 [19.31, 35.69] %, and 19.65 [14.84, 24.46] % respectively. The odds of having OSA, OS risk and SDB by the asthma patients were 2.64 [1.76, 3.52], 3.73 [2.90, 4.57] and 1.73 [1.11, 2.36] times higher (p < 0.00001 for all) in asthma than in non-asthma patients, respectively. Adult asthma patients with OSA had significantly higher BMI in comparison with asthma patients without OSA. This study reveals that the prevalence of OSA in asthma patients is considerably higher; even higher than OSA risk and SDB. Sleep studies should be performed in asthma patients with symptoms suggestive of OSA/OSA risk/SDB.

## Introduction

Asthma and obstructive sleep apnea (OSA) may coexist^1^ to result in an overlap syndrome^2^ where a bidirectional relationship may deleteriously affect each other^3^. At least 5% of the general population suffer from asthma^4, 5^; Center for Disease Control and Prevention estimates the prevalence of asthma at 7.7% (6.3% in males and 9% in females)^6^. On the other hand, OSA is an under-diagnosed condition^7^. In a general population survey, of the 451 individuals who were invited to participate in sleep study, only 3.6% had OSA diagnosis but 24% had mild, 12.5% moderate, and 2.9% had severe OSA^8^. In a larger population sample of 2121 individuals, 84% men and 61% women had mild, and 50% men and 23% women were found to have moderate OSA^9^. More recently, Senaratna *et al*.^10^ after reviewing 24 relevant studies have estimated the prevalence of OSA ranging between 9% to 38%. These authors also found this disease more common in men^11^.

Asthma is a common respiratory disorder with complex interactions between airflow obstruction, hyper-responsiveness, reversible expiratory flow limitation and inflammation^11^, whereas OSA is characterized by snoring and interruptions in breathing during sleep with symptoms such as brief paroxysmal nocturnal dyspnea, choking during sleep, and nocturia along with daytime sleep, depression and memory loss^12, 13^.

Previously, many authors have described the relationship between asthma and OSA with regards to the prevalence and risk. Asthma has been found to be an independent risk factor for the development of habitual snoring in a prospective cohort study^14^. Sleep disordered breathing has also been observed in many studies with asthma patients^15–19^. A bidirectional relationship between asthma and OSA is also evident from a study in which not only the OSA patients were reported to exhibit many asthma symptoms but also a high prevalence of asthma was reported in OSA patients^20^.

Despite the recognition of high prevalence of OSA in asthma patients, highly variable prevalence estimates are reported in the individual studies. Because this area is not systematically reviewed, no summary estimates of the association between asthma and OSA are available for clinical and/or public health implications. Keeping in view this scenario, the present study was designed to carry out a systematic review of the relevant studies and to perform a meta-analysis of the indices that could display the relationship between asthma and OSA, OSA risk and sleep disordered breathing (SDB).

## Materials and Methods

This meta-analysis is being reported in accordance with Preferred Reporting Items for Systematic Reviews and Meta-Analyses (PRISMA) statement^21^.

### Inclusion and exclusion criteria

Inclusion criterion was: clinical or epidemiological studies which examined the relationship between asthma and sleep disorders and reported the prevalence of OSA or OSA risk or SDB in asthma patients. Studies were excluded from the meta-analysis if reported only the sleep quality measures other than OSA, OSA risk or SDB, or provided qualitative information only.

### Literature search

Electronic scientific databases (EMBASE, Google Scholar, Ovid SP, PubMed/Medline, and Web of Science) were searched for the relevant research articles. The MeSH and keywords used in different logical combinations and phrases were: Asthma, wheezing, nocturnal asthma, obstructive sleep apnea (OSA), apnea, hypoapnea, Epworth sleeping scale (ESS), Apnea index (AI), hypopnea, apnea-hypopnea index (AHI), sleep disordered breathing (SDB), oxygen desaturation, forced expiratory volume (FEV), sleep efficiency, arousal index, body mass index (BMI), clinical trial, cohort, and epidemiological survey. The search encompassed original research papers published by July 2016 in online journals in English language.

### Primary and secondary endpoints

Primary endpoints were: a) the prevalence of OSA (diagnosed with sleep studies only), OSA risk (a valid questionnaire-based evaluation) and SDB (one or more abnormal breathing and/or gas exchange patterns during sleep including habitual loud snoring at least 3–4 times/week, ≥3% desaturation/hour, upper airway resistance syndrome, and central sleep apnea) in asthma patients; and b) the pooled effect size of the odds ratios of the prevalence of OSA/OSA risk/SDB between asthma and non-asthma patients. Secondary endpoints were mean differences in BMI, % predicted FEV, and ESS score between asthma patients with OSA/OSA risk/SDB and without OSA/OSA risk/SDB.

### Data extraction, synthesis and statistical analyses

Important information including outcome measures and outcomes, primary and secondary endpoints, participants’ demographic and clinical characteristics and other relevant information were obtained from the selected research articles of the respective studies and organized on datasheets. Data were extracted by two researchers independently who later cross checked the work of each other. Inter-rater reliability was good (kappa = 0.94).

Random effects meta-analyses were performed with STATA software (version 12; Stata Inc. Texas, USA) to achieve overall effect sizes of the prevalence of OSA, OSA risk, and SDB in asthma patients and to achieve a summary estimate of the odds ratio of the prevalence of OSA/OSA risk/SDB between asthma and non-asthma patients observed in the individual studies.

To assess the significance of differences in FEV (% predicted), BMI and ESS between asthma patients with and without OSA/OSA risk/SDB, meta-analyses of mean differences were carried out with RevMan software (version 5.3.5; Cochrane Collaboration) under random effects model. Between studies statistical heterogeneity was tested by tau^2^ and I^2^ indices.

## Results

**Figure 1:**
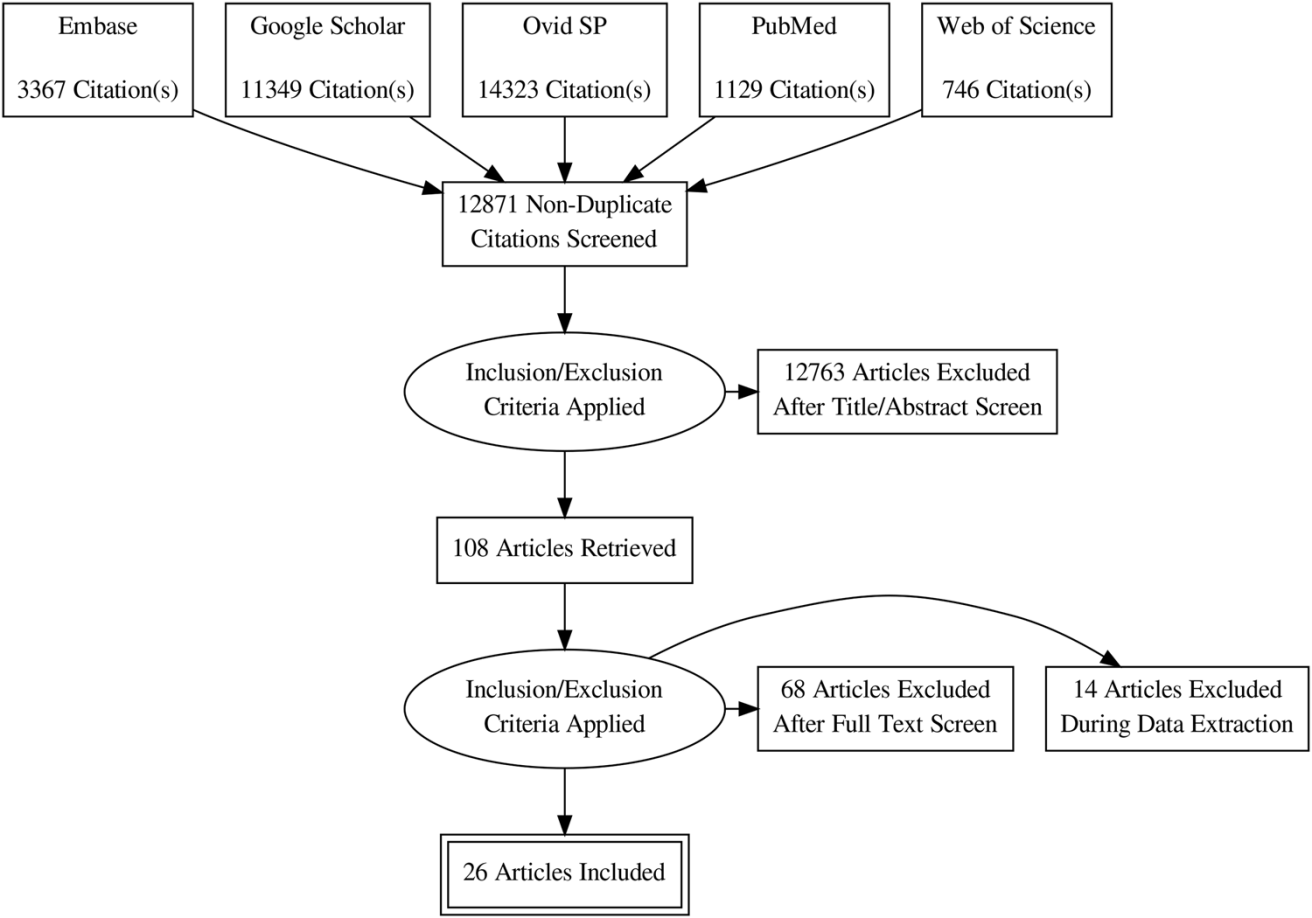
A flowchart of study screening and selection process.

Data were acquired from 26 studies^22–47^ (7675 patients) which fulfilled the eligibility criteria (Fig. 1). Important characteristics of the included studies are presented in Table S1. Average age of adult asthma patients was 48.9 ± 10 (range 42 ± 11 to 58.4 ± 15) years and that of asthmatic children 7.45 ± 3.2 (range 6.4 ± 4.4 to 9.1 ± 3.4) years. Proportion of males in this sample population was 41 ± 15% in adults and 62 ± 5.6% in children.

**Figure 2:**
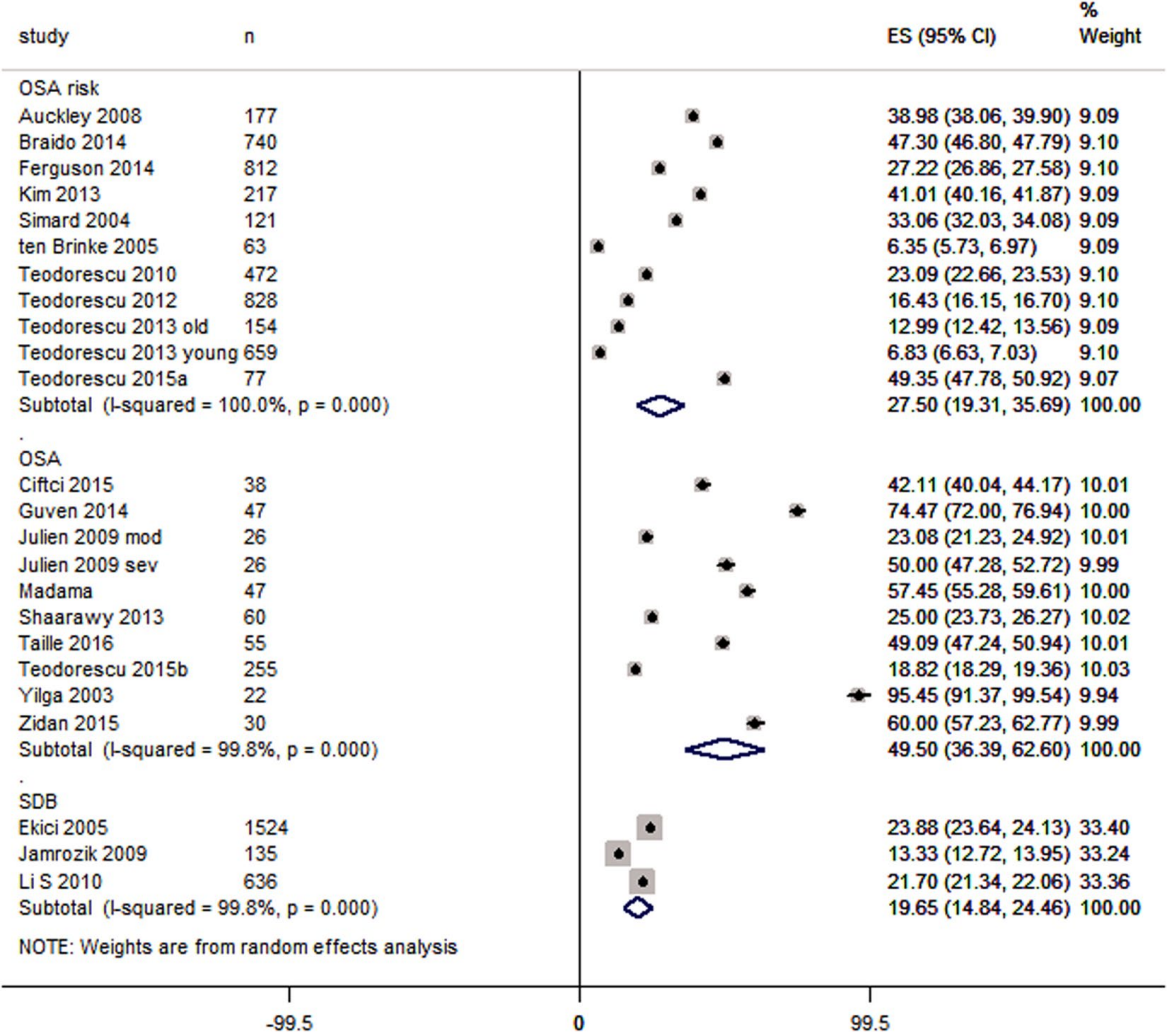
Forest graph showing the percent prevalence of OSA, OSA risk and SDB in asthma patients. Abbreviations in study identity: mo, moderate asthma, sev, severe asthma.

In adult asthma patients, the prevalence of OSA was 49.50 [36.39, 62.60] %, of OSA risk 27.50 [19.31, 35.69] %, and that of SDB was 19.65 [14.84, 24.46] % (Fig. 2). In Children, the prevalence of OSA and SDB in asthma patients was 63.04 [61.42, 64.67] % (one study data) and 22.34 [9.88, 34.79] % respectively.

**Figure 3:**
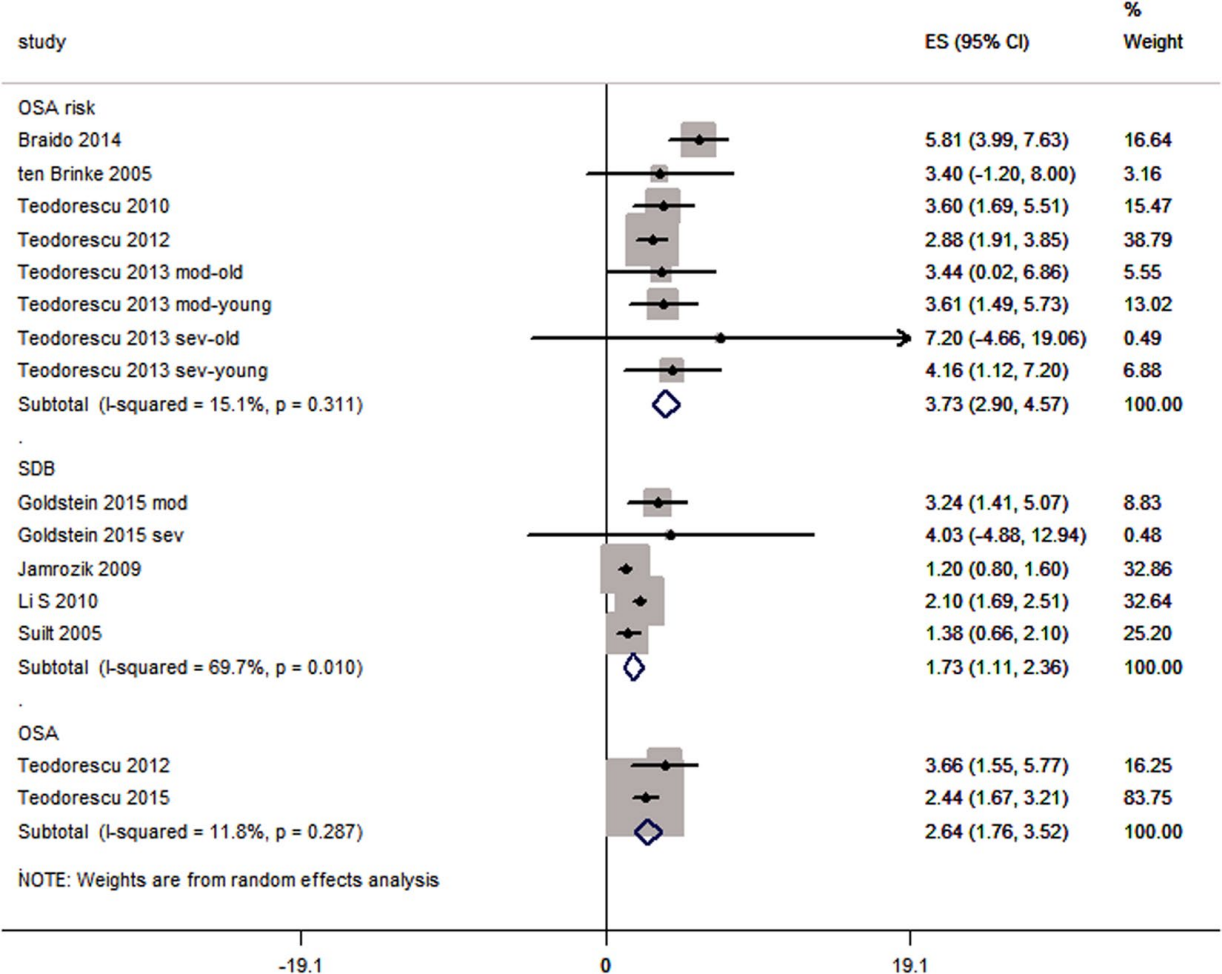
Forest graph showing the meta-analysis of odds ratios reported in individual studies with regards to OSA, OSA risk and SDB subgroups. Abbreviations in study identity: mod, moderate asthma, sev, severe asthma.

The pooled analysis of odds ratios observed in the individual studies revealed that the odds of prevalence of the OSA, OSA risk and SDB was 2.64 [1.76, 3.52] (p < 0.00001), 3.73 [2.90, 4.57]; p < 0.00001 and 1.73 [1.11, 2.36]; p < 0.00001 (respectively) times higher in asthma patients than in non-asthma patients (Fig. 3).

**Figure 4:**
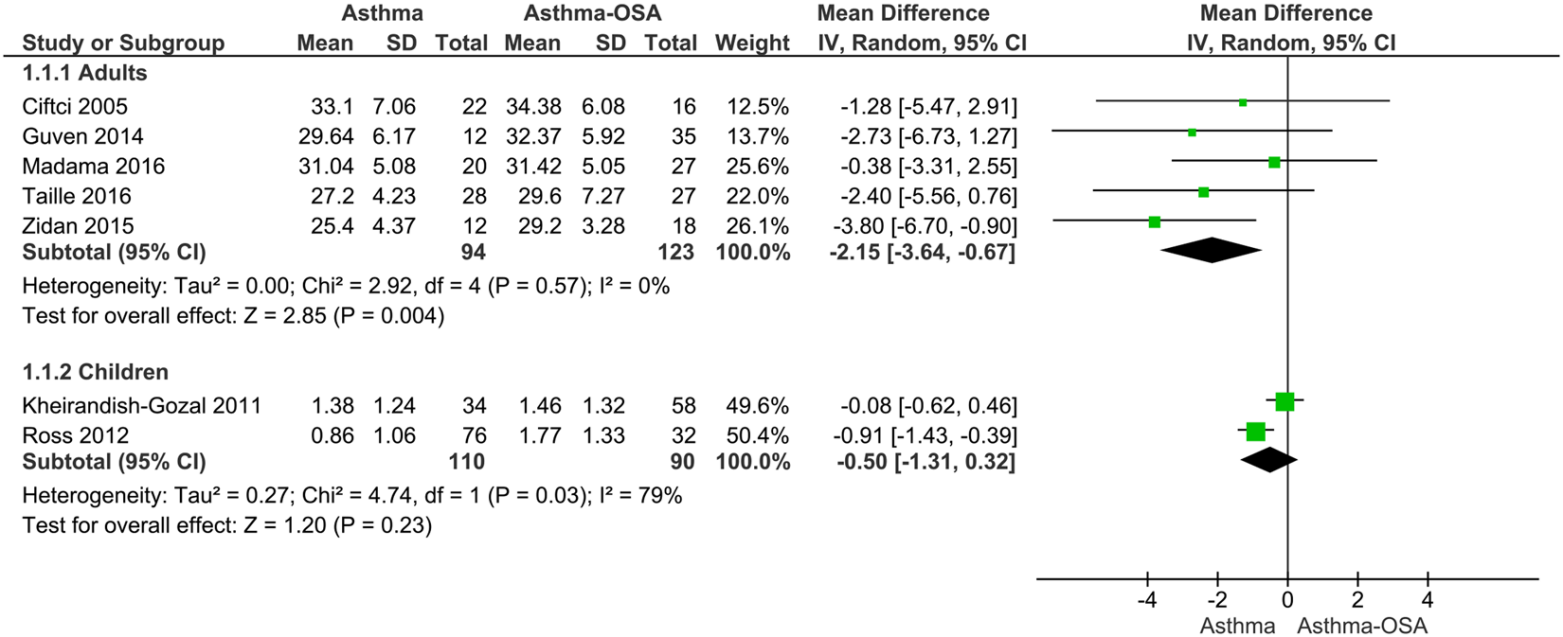
Forest graph showing the mean difference between asthma patients with OSA and without OSA in body mass index of adult and z scores of BMI in children.

Adult patients with asthma and OSA had significantly higher BMI than the asthma patients without OSA (mean difference: 2.15 [3.64, 0.67] kg/m^2^; p = 0.004; Fig. 4). In children, BMI z scores did not differ between asthma-OSA and non-asthma OSA patients. However, the number of included studies was less in this analysis (Fig. 4).

Although, there was no significant difference between the asthma patients with and without OSA in percent predicted FEV (mean difference: −2.28 [−6.79, 2.23] %; p = 0.32; Figure S1), the Epworth sleep scale score was significantly higher in the asthma patients with OSA (mean difference: 3.98 [1.30, 6.66]; p = 0.004; Figure S2) in comparison with non-OSA asthma patients.

## Discussion

This meta-analysis has revealed that the prevalence of OSA, OSA risk and SDB in adult asthma patients is 50%, 27.5% and 20%, respectively, and the odds of having OSA, OSA risk and SDB is 2.64, 3.73, and 1.73 times higher (significantly) in asthma patients than in non-asthma patients. Asthma patients with OSA also had significantly higher BMI in comparison with non-asthma patients.

Both asthma and OSA have airway obstruction in the pathogenesis and have many diurnal and nocturnal symptoms in common^48^. Obstructive sleep apnea is identified as an independent risk factor for asthma exacerbation^40^, and OSA is reported to be more prevalent among patients with severe asthma than in moderate asthma which may be linked to the potential pathophysiologic interaction between OSA and asthma severity^30^. Moreover, a significant alleviation in asthma symptoms has been reported with the long-term use of continuous positive air pressure (CPAP) in patients with both asthma and OSA^49, 50^.

Obesity and related comorbidities, including SDB and gastro-esophageal reflux are found to be highly prevalent in asthma patients in many epidemiological studies^31, 35, 51^. A high body mass index may be a worsening factor in both conditions^52^. Although, obesity is a well-recognized risk factor for asthma in adult patients, but a causal relationship is still lacking^53^. There is also some evidence to suggest that hypothyroidism may also play a role in obesity mediated OSA severity^54^.

An important aspect that needs to be further studied is the association between AHI and asthma severity. In the included studies of present meta-analysis, some observations indicate that there exists a positive relationship between SDB and asthma severity e.g. Goldstein *et al*.^27^ mentioned that percentage of snoring patients was 33%, 38%, and 43% in mild, moderate, and severe asthma patients, respectively. Ross *et al*.^35^ also found that percentage of patients with snoring and desaturation was higher in severe asthma (55%) than in moderate asthma (20%) patients. Julien *et al*.^30^ found that sleeping efficiency and arousal index were significantly higher in severe than in moderate asthma patients. Zidan *et al*.^47^ found that percentage of OSA-asthma patients was 5.6% in well-controlled asthma patients, 61% in partially controlled and 33.3% in uncontrolled asthma patients.

Based on the above-mentioned findings, it can be presumed that the relationship between OSA and asthma have therapeutic implications. Effective treatment of OSA can favorably impact asthma control; CPAP is one such treatment^49, 50^. The CPAP therapy is also found to reduce gastroesophageal reflux disease and inflammation in OSA patients which may be beneficial for both asthma and OSA. However, CPAP has also been reported to increase bronchial hyper-responsiveness in non-asthma OSA patients^55, 56^ and cause sleep abnormalities in non-OSA asthma patients^57^ which makes it necessary to further investigate the effectiveness and consequences of this therapy.

Asthma-OSA overlap appears to be an area where patient-centered healthcare is more important because not only asthma is associated with significantly more comorbidities in comparison with other diseases^58^ but it is also evident that OSA has relationship with obesity and hypothyroidism^54^. Therefore, consideration of relevant therapies may have better impact e.g. bariatric surgery is found to be associated with improvement in symptoms of OSA and asthma^37^. Vitamin D deficiency has been reported to be associated with OSA severity^59^ and vitamin D treatment in asthma patients is found to reduce exacerbations^60^. Thus, it seems imperative that while managing asthma patients, physicians should examine the symptoms suggestive of OSA and consider differential diagnostics, especially in unstable, under-controlled and overweight patients that may also help in choosing a therapeutic strategy.

## Conclusion

The prevalence of OSA in adult asthma patients is estimated at 50% in this meta-analysis and the odds of having OSA is 2.64 times higher in asthma patients than in the non-asthma patients. Moreover, the prevalence of OSA risk and SDB are estimated at 27.5% and 20% respectively. The odds of having OSA risk and SDB is 3.73 and 1.73 time higher (respectively) in asthma patients than in non-asthma patients. The prevalence of OSA was higher than the prevalence of SDB in asthma patients which indicates the existence of a bilateral relationship between OSA and asthma to result in a more severe phenotype. Asthma patients with OSA also had significantly higher BMI in comparison with non-OSA asthma patients. Therefore, a closer look at the symptoms suggestive of OSA is necessary in asthma patients especially in more severe forms and overweight individuals.

### Statement of Ethics

This study does not involve ethical review by virtue of its design.

## Electronic supplementary material

Supplementary info
