# Supplementary material for: Association of Obstructive Sleep Apnea with Asthma: A Meta-Analysis
Source: Sci Rep. 2017 Jun 22;7:4088. doi: 10.1038/s41598-017-04446-6 (PMC5481327; doi:10.1038/s41598-017-04446-6)
Supplement: Supplementary file 1 — Supplementary info [file 41598_2017_4446_MOESM1_ESM.pdf]

Title Page

Association of Obstructive Sleep Apnea with Asthma: A Meta-Analysis

De-Lei Kong[1,*], MD; Zheng Qin[1], MD; Hui Shen[1], MD; Hong-Yu Jin[1], MD; Wei Wang[1], MD, Zan-Feng Wang[1], MD

[1]Department of Respiratory Disease, The First Affiliated Hospital of China Medical University, Shenyang, Liaoning 110001, China

[*]Corresponding author:

Dr. De-Lei Kong

Department of Respiratory Disease, The First Affiliated Hospital of China Medical University

155 Nanjing Northern St, Heping District, Shenyang, Liaoning 110001, China

Tel: +86-24-83283195, Fax: +86-24-83282002

Email: kongdelei_med@126.com

Running title: Obstructive sleep apnea and asthma

Table S1: Important characteristics of the included studies						
**Study**	**n**	**Diagnosis tool**	**Design/patients**	**Age** ± sd	**BMI** ± sd	**% males**
Auckley 2008	177	Berlin Questionnaire	Prospective/consecutive series	48±14		26.55
Braido 2014	740	STOP-BANG	Clinical survey/physician recruited	48.2±15.2	25.9±4.6	58
Ciftci 2015	38	PSG	Clinical study/Nocturnal asthma	45±8		73.68
Ekici 2005	1524	RSHQ	Epidemiological survey		27±4.6	
Ferguson 2014	812	SA-SDQ	Cross sectional study/routine	±4614	29±6.8	33
Goldstein 2015	263	PSQ	Cross sectional study/routine	6.4±4.4		62.35
Guven 2014	47	PSG	Cross sectional study/difficult-to-treat	48.7±9.45	31.67±6.04	19
Jamrozik 2009	135	Questionnaire	Cross sectional survey	46±14	24.9±3.9	39.26
Julien 2009 mod	26	PSG	Clinical study/difficult-to-treat asthma	48.4±1.8	27.8±1.2	46.15
Julien 2009 sev	26	PSG	Clinical study/difficult-to-treat asthma	48.4±1.8	27.8±1.2	53.84
Kheirandish-Gozal 2011	92	PSG	Prospective/consecutive series	6.85±1.8		53
Kim 2013	217	QLQA	Prospective/consecutive series	58.4±15.4		42
Madama 2016	47	PSG	Retrospective/routine	55.56±13	31.36±5.33	32
Ross 2012	108	Questionnaire	Prospective/consecutive series	9.1±3.4		67.59
Shaarawy 2013	60	PSG	Cross sectional study/difficult-to-treat	46±13	30±6.1	25
Simard 2004	121	Questionnaire	Cross sectional study/comorbid	51.4±1.8		
Sulit 2005	121	Questionnaire	Cohort study/community-based	9.5±0.8	18.9±4.6	64.46
Sulit 2005	121	Questionnaire	Cohort study/community-based	9.5±0.8	18.9±4.6	64.46
Taille 2016	55	PSG	Prospective/severe asthma	47.8±1.7	28.4±0.8	22
ten Brinke 2005	63	PSG/Questionnaire	Cross sectional/Difficult-to-treat Asthma	41.5±14.1		26.98
Teodorescu 2010	472	SA-SDQ	Prospective/consecutive series	47±14	28.4±6.4	36
Teodorescu 2012	828	SA-SDQ	Prospective/consecutive series	47±14	29±7	33
Teodorescu 2013 old	154	SA-SDQ	Prospective/consecutive series	66±4	29±6	38
Teodorescu 2013 young	659	SA-SDQ	Prospective/consecutive series	42±11	29±7	32
Teodorescu 2015a	77	SA-SDQ	Prospective/consecutive series	50.4±7.7	28.9±5.5	48
Teodorescu 2015b	255	PSG	Prospective epidemiological survey	39±13	31±8	40
Yilga 2003	22	PSG	Prospective cohort/difficult-to-treat			
Zidan 2015	30	PSG	Prospective/consecutive series	49.12±3.43		43.33

Study or Subgroup	Asthma-OSA Mean	Asthma-OSA SD	Asthma-OSA Total	Asthma Mean	Asthma SD	Asthma Total	Weight	Mean Difference IV, Random, 95% CI
Ciftci 2005	70.25	21.17	16	65.52	17.37	22	8.4%	4.73 [-7.93, 17.39]
Guven 2014	64.43	20.58	35	58.17	20.76	12	7.6%	6.26 [-7.32, 19.84]
Kheirandish-Gozal 2011	82.1	7.1	58	87.9	6.4	34	22.6%	-5.80 [-8.62, -2.98]
Ross 2012	90.9	17.1	32	98.7	17.7	76	15.3%	-7.80 [-14.94, -0.66]
Taille 2016	69.9	4.2	27	67.4	4.5	28	23.3%	2.50 [0.20, 4.80]
Zidan 2015	72.8	4.8	18	78.2	2.62	12	22.8%	-5.40 [-8.07, -2.73]
**Total (95% CI)**			**186**			**184**	**100.0%**	**-2.28 [-6.79, 2.23]**

Heterogeneity: Tau² = 21.36; Chi² = 32.82, df = 5 (P < 0.00001); I² = 85%
Test for overall effect: Z = 0.99 (P = 0.32)

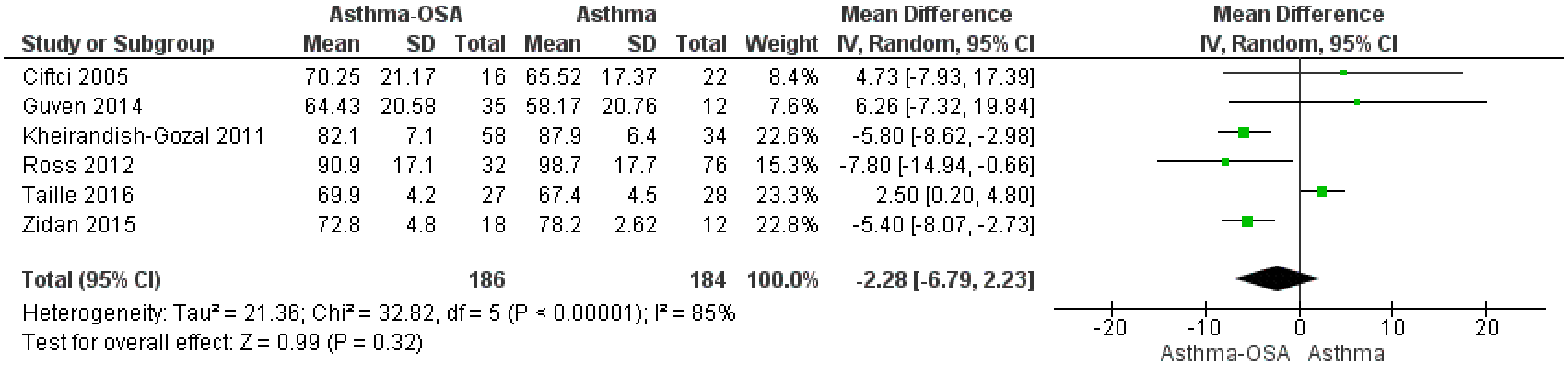

Figure S1: Forest graph showing the mean difference between asthma patients with OSA and without OSA in percent predicted FEV.

Study or Subgroup	Asthma-OSA Mean	Asthma-OSA SD	Asthma-OSA Total	Asthma Mean	Asthma SD	Asthma Total	Weight	Mean Difference IV, Random, 95% CI
Guven 2014	9.57	2.98	35	3.75	2.34	12	39.4%	5.82 [4.17, 7.47]
Madama 2016	11	6.2	27	11	6.6	20	24.5%	0.00 [-3.72, 3.72]
Zidan 2015	10.22	3.7	18	5.54	2.15	12	36.1%	4.68 [2.58, 6.78]
**Total (95% CI)**			**80**			**44**	**100.0%**	**3.98 [1.30, 6.66]**

Heterogeneity: Tau² = 4.04; Chi² = 7.87, df = 2 (P = 0.02); I² = 75%
Test for overall effect: Z = 2.91 (P = 0.004)

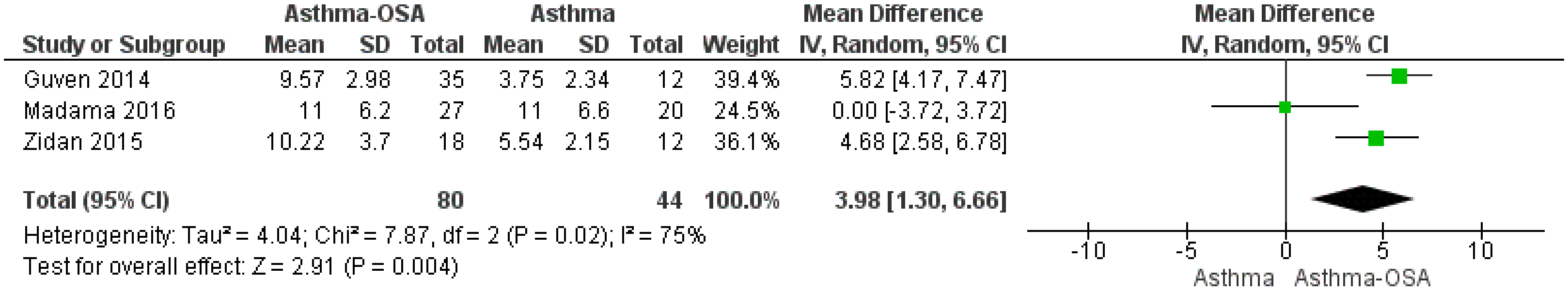

Figure S2: Forest graph showing the mean difference between asthma patients with OSA and without OSA in Epworth sleep scale scores.
